# Supplementation stocking of Lake Trout (*Salvelinus namaycush*) in small boreal lakes: Ecotypes influence on growth and condition

**DOI:** 10.1371/journal.pone.0200599

**Published:** 2018-07-12

**Authors:** Olivier Morissette, Pascal Sirois, Nigel P. Lester, Chris C. Wilson, Louis Bernatchez

**Affiliations:** 1 Institut de Biologie Intégrative des Systèmes (IBIS), Université Laval, Quebec City, Quebec, Canada; 2 Chaire de recherche sur les espèces aquatiques exploitées, Laboratoire des sciences aquatiques, Département des sciences fondamentales, Université du Québec à Chicoutimi, Chicoutimi, Quebec, Canada; 3 Aquatic Research and Monitoring Section, Ontario Ministry of Natural Resources and Forestry, Peterborough, Ontario, Canada; 4 Aquatic Research and Development Section, Ontario Ministry of Natural Resources and Forestry, Peterborough, Ontario, Canada; Southwest University, CHINA

## Abstract

Supplementation stocking is a commonly used management tool to sustain exploited fish populations. Possible negative consequences of supplementation on local stocks are a concern for the conservation of wild fish populations. However, the direct impacts of supplementation on life history traits of local populations have rarely been investigated. In addition, intraspecific hybridization between contrasting ecotypes (planktivorous and piscivorous) has been seldom considered in supplementation plans. Here, we combined genetic (genotype-by-sequencing analysis) and life history traits to document the effects of supplementation on maximum length, growth rates, body condition and genetic admixture in stocked populations of two Lake Trout ecotypes from small boreal lakes in Quebec and Ontario, Canada. In both ecotypes, the length of stocked individuals was greater than local individuals and, in planktivorous-stocked populations, most stocked fish exhibited a planktivorous-like growth while 20% of fish exhibited piscivorous-like growth. The body condition index was positively related to the proportion of local genetic background, but this pattern was only observed in stocked planktivorous populations. We conclude that interactions and hybridization between contrasting ecotypes is a risk that could result in deleterious impacts and possible outbreeding depression. We discuss the implications of these findings for supplementation stocking.

## Introduction

The voluntary introduction of exogenous animals and plants is one of the most frequent anthropogenic perturbations of wild populations [1]. Deliberate releases of exogenous fish commonly have the goal of increasing the abundance of a threatened population, increasing potential fish harvest or introducing new species [2]. For decades, the stocking of lakes with fish reared in hatcheries has been an important management approach, both in North America and Europe [3, 4]. Stocking practices are diverse and are classified according to a variety of terminologies (e.g., put-and-take, conservation or fishery enhancements) generally based on the specific objectives of the stocking program [5, 6]. Among them, supplementation stocking (or fishery enhancement) aims to compensate for low productivity or a reduction in the abundance of populations that have been either (over-)exploited by recreational fisheries or perturbed by anthropogenic disturbances [7].

Supplementation contributes to the maintenance of economically and socially important fish populations—largely salmonids in North America—targeted by angling activities [4, 6]. Although supplementation stocking is an effective management strategy in some circumstances, the potential of direct and indirect negative impacts on wild populations remains hotly debated [8–11]. The short- and long-term consequences of this approach on native fish populations are not fully understood, and there is a sustained interest to better predict the ecological, genetic and evolutionary impacts of stocked fish introduced into natural systems [12, 13].

A main concern regarding supplementation stocking is the ecological and genetic interactions between stocked and local individuals [5]. Depending on the species, enhancement of top-predator demographics can destabilize local food webs [14]. Furthermore, the addition of stocked individuals may induce deleterious density-dependent phenomena in local populations [15] or increase the risk of disease transmission [16]. Intraspecific phenotypic and/or genotypic differences can develop rapidly between introduced hatchery-reared fish and local stocks through inadvertent or deliberate selection [17–19] and/or epigenetic modifications [20]. Differences between hatcheries and natural water bodies in terms of environmental conditions and selection pressure may favor the survival of atypical individuals, the emergence of maladapted predator avoidance or agonistic behavior [21] that could directly alter natural ecological processes [22, 23].

Introgressive hybridization between local and hatchery-reared individuals can also have genetic impacts on local populations [11, 24–26]. The consequences of introgression are generally negative and include a decreased effective population size [1, 27], alteration of genetic integrity [28], changes in gene expression [29], loss of local adaptations and a reduction of fitness [1, 9, 11, 30]. However, supplementation with native broodstocks or with stocks sharing a high similarity with the recipient populations has demonstrated few or no deleterious effects from introgressive hybridization [13, 31, 32]. After several generations, local selection may purge foreign genes from the wild populations [10, 33, 34].

Compared to the plethora of studies documenting genetic changes subsequent to supplementation [26, 32], few studies have documented the impact of introgressive hybridization on the life history traits of local populations (e.g., growth, survival and reproduction) [24, 29]. Growth of domesticated and wild fish has often been compared within controlled and semi-controlled systems [4, 35], but fewer studies have compared the growth of supplemented fish and their hybrids in natural settings [23, 36]. Growth in fish is a complex process that involves gene × environment interactions influenced by multiple exogenous factors, including water temperature, prey availability and population density. Thus, growth is best studied in a natural setting [37, 38]. Modification of growth can be of prime importance as growth rates and length-at-age influence fish survival [39, 40], reproductive success [41] and competitive capacity [42, 43]. Growth rate is thus one of the three vital rates (along with recruitment and mortality) linked to the productivity of fish populations and can be viewed in some cases as a proxy for reproductive success [44].

Lake Trout (*Salvelinus namaycush*) is a large (mean: 400–500 mm), long-lived (up to 45 years) and late maturing (with intermittent spawning) salmonid found in deep and cold freshwater lakes in North America. It is one of the most sought-after species by recreational anglers. The species’ natural distribution matches closely the limits of Pleistocene glaciation, and numerous isolated populations are scattered throughout the Boreal Shield ecozone [45]. Across its native range, Lake Trout exhibits marked variations in life history [46]. Notoriously, three Lake Trout ecotypes (i.e., lean, siscowet and humper) have been shown to differ in terms of diet, size, morphology and ecology within the Laurentian Great Lakes, Great Slave Lake [47] and Mistassini Lake [48]. Even in smaller lakes and at a smaller geographic scale, populations can exploit a variety of divergent ecological niches, primarily attributed to local environmental conditions and the available prey community. For instance, Rasmussen et al. [49] described three classes of small lakes (e.g., excluding large North American lakes) that host Lake Trout populations, classified according to the presence/absence of different prey types. These categories reflected important differences in local conditions that translated into marked differences in Lake Trout life history including growth rate, diet, age at maturity, age structure and spatial distribution [45, 50–53]. The various life histories exhibited among Lake Trout populations can be summarized into two common ecotypes (e.g., planktivorous and piscivorous) typical of small boreal lakes. Ecotypes reflect the combined influence of environmental conditions, niche availability and genetics [51, 54]. The planktivorous ecotype is characterized by low growth rates, early maturation (~ 6 years) and a shorter maximum length of fish (< 450 mm), whereas piscivorous ecotypes exhibit high growth rates, late maturation (> 9 years) and a larger (> 600 mm) maximum length of fish [54, 55]. Some particular lakes can host both ecotypes living in sympatry, but the vast majority host a single allopatric ecotype.

The stocking of Lake Trout has been used for population supplementation for over a century [56]. In Quebec, Canada, 46% of lakes in the southern portion of the province that harbor a Lake Trout population exploited for angling have been stocked at least once since 1928 [57]. Stocking in Quebec lakes has been documented since 1900; recorded information includes the source populations, the number of stocking events, the numbers of stocked individuals and the life stages at stocking. These records demonstrate that the ecotypes of source and recipient populations have never been considered in stocking practices. Supplementation in Quebec always uses captive-reared broodstock from wild breeders that originate from allopatric Lake Trout populations of the piscivorous ecotype. This stocking approach has been used even when the recipient populations were allopatric populations of a planktivorous ecotype. In the most detailed study of the genetic impacts of stocking on wild populations of Lake Trout in boreal lakes, Valiquette et al. [34] showed that stocking increased intra-lake genetic diversity, but also decreased inter-lake genetic distance among stocked populations (i.e., genetic homogenization). They also observed a positive relationship between the number of stocking events and the proportion of admixed individuals. Evans et al. [58, 59] also reviewed other impacts of stocking on Lake Trout populations. However, to our knowledge, the impact of stocking on fish growth and condition involving different ecotypes has never been documented.

This study aims to determine the impacts of Lake Trout ecotype on the genetic and phenotypic effects of supplementation stocking. Specifically, we assess whether stocking a piscivorous ecotype into recipient populations of piscivorous and planktivorous ecotypes has direct or indirect effects on fish growth. We compare individual Von Bertalanffy growth model parameters of fish having local, stocked and hybrid genetic origins. We also test the hypothesis that direct (i.e., growth of stocked individuals) and indirect effects (due to hybridization) are more important when stocking involves an ecotype different from that of the recipient population (here, a planktivorous ecotype). Ultimately, we aim to evaluate outcomes of supplementation stocking to propose strategies that could minimize potential negative impacts.

## Methods

### Study design

This study is based on a hierarchical design with two factors: ecotypes of populations (levels: piscivorous and planktivorous) and stocking history (levels: stocked and unstocked) using lakes as replicates. The selected lakes are located in the boreal ecozone of Quebec and Ontario, Canada, are similar in size, share similar abiotic conditions (Table 1) and harbor a single—piscivorous or planktivorous—Lake Trout population. These trout ecotypes were determined based on the presence or absence of pelagic forage fish and the maximum size of Lake Trout as determined from governmental surveys (Ministère des Forêts, de la Faune et des Parcs du Québec (MFFP), unpublished data). We selected stocked lakes based on i) known stocking history (MFFP, unpublished data; S1 Table) to represent populations stocked at least once in the last twelve years and having a stocking history longer than 20 years, and ii) genetic information of the extent of introgressive hybridization obtained in a previous study [34]. As such, we wanted to maximize the probability of finding individuals having local or stocked origin genotypes and their hybrids.

**Table 1:** Study design and key information for each lake including latitude/longitude, the year of most recent stocking, lake area, average annual air temperature and design treatment groups.

Lake	Lat.	Long.	Last year stocked	Lake area (ha)	Mean air temp. (°C)	Design treatment
Desert	46.573	-76.322	–	329	3.08	Unstocked–Piscivorous
Marguerite	47.029	-75.804	–	622	2.34	Unstocked–Piscivorous
Opeongo	45.688	-78.363	–	5154	NA	Unstocked–Piscivorous
Antoine	46.370	-76.986	–	435	3.33	Unstocked–Planktivorous
Bondy	47.083	-75.851	–	531	2.18	Unstocked–Planktivorous
Shirley	45.689	-78.128	–	503	NA	Unstocked–Planktivorous
Cayamant	46.106	-76.277	2011	725	3.93	Stocked–Piscivorous
Cèdres	46.305	-76.111	2011	282	3.84	Stocked–Piscivorous
Louisa	45.772	-74.417	2006	440	3.98	Stocked–Planktivorous
McFee	45.715	-75.623	2001	93	4.03	Stocked–Planktivorous

#### Stocking history

Stocking has generally used first-generation progeny (F1) of wild breeders, captured from known spawning sites in source lakes (e.g., Blue Sea and Trente et Un Milles lakes). Eggs are artificially fertilized in hatcheries and progeny reared in until stocking. Age at stocking varies between a few months (fry stage) to more than a year (1+ year). Stocking densities are adjusted by the area of the stocked lakes and vary over time based on angling exploitation levels and previously measured catch per unit effort (MFFP, pers. comm). Neither domesticated strains nor adult fish have been used in the stocking of the study lakes.

### Fish sampling and phenotype scoring

**Fig 1:**
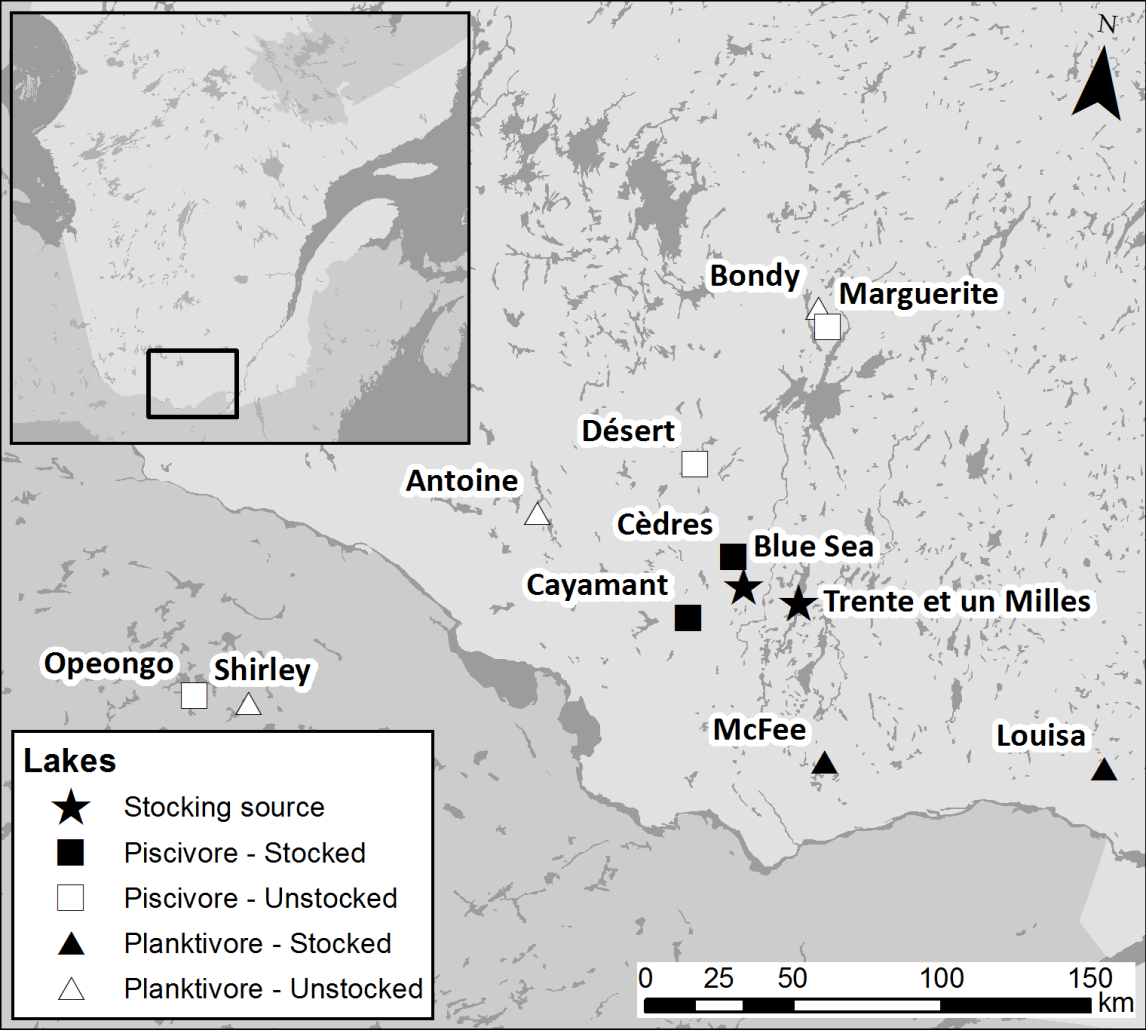
Lake Trout sampling locations with stocking source lakes (solid stars). Unstocked (open symbols) and stocked lakes (solid symbols) had either piscivorous ecotype (square) or planktivorous ecotype (triangle) Lake Trout populations.

Fish were collected in 2013 using an experimental gill netting method following the MFFP standard Lake Trout sampling protocol (S1 Appendix). Sampling occurred in collaboration with the MFFP and the Ontario Ministry of Natural Resources and Forestry (MNRF), both using the same sampling protocol. Experimental gill nets used during sampling were designed and installed to ensure representative catches of the population’s length classes. The MFFP also provided 30 fish from each of two stocking source populations (Blue Sea and Trente et Un Milles lakes) (Fig 1).

We assessed sex and sexual maturity by visual inspection of gonads; maturity was recorded as a binary factor (immature, mature). Immediately upon capture, we measured total length (TL, mm) for every fish. Weight (g) was measured on fresh fish using a portable digital scale. A biopsy of the adipose fins (or pectoral fin if the adipose was missing) was taken from each fish and stored in 95% ethanol in individual plastic vials (Eppendorf, Mississauga, ON). Sagittal otoliths were extracted from the fish using plastic forceps. The otoliths were cleaned with water, dried and stored in Eppendorf plastic vials.

#### Genotype-by-sequencing analyses

Genomic DNA extraction followed the salt extraction protocol [60] in conjunction with the RNAase A (Qiagen) procedure, following manufacturer specifications. We assessed DNA quality by noting the presence of smears and poor-quality samples on a 1% agarose gel. DNA samples of poor quality were re-extracted or discarded from further analysis. DNA quantity was estimated using a NanoDrop spectrophotometer (Thermo Scientific) and then a Quant-iT Picogreen dsDNA Assay Kit (Invitrogen). Genotyping-by-sequencing (GBS) libraries were prepared with the *PstI* and *MspI* restriction enzymes following a modified version of a two-enzyme GBS protocol used in a previous Lake Trout study [61]. A total of 48 individuals were barcoded and pooled per library, and we used 96 barcode sequences of four to eight nucleotides. Real-time PCR was used to quantify libraries. Single-end 100-bp-length sequencing on the Illumina HiSeq2000 platform was conducted at the Genome Quebec Innovation Centre (McGill University, Montreal, QC).

#### Bioinformatics

Adapters were removed from the sequencing raw data with cutadapt v 1.8.2 software in single-end mode [62]. Individual sequences within each library were demultiplexed and trimmed at 80 bp using *process_radtag*s in STACKS v 1.35 [63]. Reads were aligned and potential de novo polymorphic loci were built using a minimum stack depth coverage of three (*m* = 3) and a maximum of two nucleotide mismatches (*M* = 2) in the *ustacks* module (within STACKS). A catalog of single-nucleotide polymorphisms (SNPs) was assembled using *cstacks* with default parameters. Genotyping was achieved using *sstacks* to match individual reads against the catalog loci.

#### Loci quality and filtering

The *populations* module of STACKS (*r* = 0.5, *m* = 3) and a subsequent quality filtering process of SNP markers were applied to either pairs of source-target populations or, in one case, trios (Louisa Lake was stocked once by Blue Sea sources in 1998 and Trente et Un Milles, otherwise *p* = 1 or 2, respectively). We filtered out loci having too low a coverage (*m* < 5), a maximum allele coverage of 30 and maximum heterozygosity up to 0.7. We only retained markers that were present in at least 70% of the individuals within a population. Loci filtering also included genomic likelihood, minor allele frequency and allelic imbalance. Finally, using the *summary_haplotype* function implemented in the R package stackr [64], we filtered loci for sequencing artifacts and paralogs by removing loci having more than two alleles. The filtering procedure using the STACKS software is part of a freely available workflow on GitHub (https://github.com/enormandeau/stacks_workflow). All module parameters are listed in the supplementary materials (S2 Table).

### Individual assignment

Individual admixture proportions (*Q*) were estimated for stocked populations using the Bayesian clustering method implemented in the software ADMIXTURE v 1.3 [65]. The assumed number of clusters was either *K* = 2 or *K* = 3 according to stocking history data, but an extended range of probable *K* was also tested (*K* = 1 to 6). The best number of clusters (*K)* in each analysis was inferred using cross-validation error. Individual admixture proportion’s standard error (SE) was estimated from 2000 bootstrapped replicates. Stocked fish were assigned when *Q*_*stocking source*_ + SE ≥ 90%, local fish when *Q*_*stocking source*_ + SE ≤ 10%, whereas others were classified as hybrids (*Q* values ranging from 10 to 90%).

### Age, growth and condition of fish

#### Individual age estimation

Age estimation and growth models were based on otolith annuli counts and measurements. The right sagittal otolith was embedded in a two-part epoxy resin (Miapoxy 100, Freeman, OH, USA) and cut in 1-mm-thick traversal sections with a slow-speed diamond-bladed saw (IsoMet saw; Buehler, IL, USA). After sectioning, otolith microstructure contrasts were amplified by grinding and polishing with sandpaper (2000 grit Wetordry^TM^, 3M^TM^) and aluminum oxide lapping film (1- and 5-μm lapping film, 3M^TM^). Sagittal sections were mounted onto a microscope slide using thermoplastic glue (Crystalbond^TM^ 509; Aremco^TM^ Products Inc., NY).

Digital images of each otolith were captured using a digital camera (Leica DMC) coupled to a dissection microscope (Leica MZ12) at a 30–60x magnification. Images were captured on a black background with direct and reflected light. Age counts and increment measurements (μm) were calculated from the nucleus to the maximum ventral radius of the otolith following established methods and criteria [66, 67]. Two independent readings (two readers) using ImageJ v 10.2 software measured the annual increments of otoliths [68]. We performed a third additional count when the first counts were not concordant. A total of 400 of 476 captured fish had otoliths suitable for age determination (16% rejected). Twenty-nine percent of the otolith measurements required a third age count due to initial results not agreeing.

#### Modeling the population effects of stocking

We modeled the effects of ecotype and stocking history on estimated age and measured TL (response variables) in stocked and unstocked populations using mixed effect models. Factors of the model were ecotypes (fixed, two levels: piscivorous and planktivorous), stocking history (fixed, two levels: stocked and unstocked) nested within ecotypes and sex (fixed, two levels: male and female). Populations (lakes) were treated in the model as a random factor. We assumed that sampled individuals were representative of populations and only the ecotype affected measured TL. Linear mixed models ran using the function *lme* in the R package *nlme*.

#### Length-at-age back-calculations

A regression of TL as a function of otolith radius at capture was fit using a power function model (L = u*S^*v*^), where *S* is the otolith radius at capture and *L* is TL at capture. The regression used a natural log-transformation: ln(L) = v*ln(S) + ln(u). For every sampled fish, radial measurements of annuli increments were used to back-calculate length-at-age using the body proportional hypothesis (BPH) equation: *L*_*i*_ *= [(S*_*i*_*)/(S)]*^v^*L where *L*_*i*_ is the back-calculated length-at-age *i*, *S*_*i*_ is the radial measurement (μm) of the annulus and *v* is calculated by regression [69].

#### Calculation of growth parameters

We estimated population-specific and individual growth parameters using typical Von Bertalanffy growth models (VBGM) fit with a non-linear regression technique on the back-calculated length-at-age of fish from each population (and individual fish). This was performed using the *nls* function implemented within the FSA R package [70]. This model *L*_*t*_ *= L*_*inf*_ *[1 –e*^*-K(t-t0)*^*] + ε* describes back-calculated length (*L*_*t*_) as a function of asymptotic length (*L*_*inf*_), the Von Bertalanffy growth parameter (*K*), the theoretical length-at-age 0 (*t0*) and additive process error (ε). The Ford-Walford method (function *vbstart*) estimated the initial values of parameters (*L*_*inf*_, *K* and *t0*) and bootstrapping (999 iterations) to estimate the parameters and their SE.

As in Hansen et al. [71], we removed individual growth parameter estimates having a SE exceeding 25% of the estimate from subsequent analyses (a total of 17 fish). We assumed that this SE value was indicative of a poor fit of the model. Removing this data precludes the inclusion of outliers that would lead to an invalid interpretation of the biological significance of observed patterns. Comparison of individual VBGM parameters is advantageous as it tests growth rate and length on a single scale, independent of the age of fish at the time of capture. However, to best represent reality, individual models need high fit quality. Omega (ω) growth rate was calculated for each individual as *ω = L*_*inf*_ * K, as suggested by Gallucci & Quinn [72]. This parameter is the slope of the growth curve at its origin measured in mm/year. It can be biologically interpreted as the growth rate early in life. Omega has important advantages over the Von Bertalanffy growth coefficient (*K*), as it is easily interpretable (growth rate, unit mm/year) and is independent of *L*_inf_, thus offering a better statistical robustness [72].

#### Calculation of the body condition index

The relative weight condition index (*Wr*) as Wr = 100 * (W/Ws) represented the individual body condition index, where *W* is the weight of captured fish and *Ws* is the standard weight, calculated from a species-specific equation as first suggested by Wege and Anderson [73]. They suggested that *Wr* relates not only to “fish plumpness” but also the general health of a fish when calculated from a standard equation that encompasses the larger possible variability of the target species [74]. We calculated standard weight for every fish based on their TL and the published equation derived from 58 Lake Trout populations from throughout their native range of distribution: *log*_*10*_*Ws = -5*.*681 + 3*.*2462 log*_*10*_*TL* [75].

As piscivorous and planktivorous ecotypes may exhibit slightly different body shapes [54], we tested whether *Wr* could be compared among and within ecotypes. We compared the *Wr* of unstocked populations between ecotypes using a Student’s *t*-test with the Welch estimation of the degree of freedom for unequal sizes, under the assumption that the unstocked populations were representative of locally adapted populations and should therefore have comparable values (≈ 100) irrespective of the population’s ecotype. Finally, the impact of length and age on *Wr* values was also assessed through linear regressions with these data.

#### Modeling of the effect of stocking on growth parameters

We modeled the effects of genetic background on individual Lake Trout growth parameters (*L*_*inf*_, ω) and the condition index (*Wr*) for each stocked population. Again, we used linear mixed effect models with factors of the model being ecotypes (fixed, two levels: piscivorous and planktivorous), genetic origin (fixed, three levels: local, hybrid and stocked) nested within ecotype, sex (fixed, two levels: male and female), and population (lake) was treated as a random factor. Linear mixed models were run using the function *lme* in the R package *nlme*.

We also evaluated the effects of introgressive hybridization on *Wr* in stocked populations via the linear regression of *Wr* as a function of the proportion of local assignment (*Q* values) from the ADMIXTURE analysis using a dataset organized by ecotypes.

## Results

Age estimates of all sampled Lake Trout varied between 4 and 28 years (mean = 12 years, SD = 3.45; Table 2). Measured TL varied between 195 and 862 mm (mean = 435 mm, SD = 104.93). Stocking history and ecotype effects on the estimated age and TL showed a significant negative impact of the planktivorous ecotype on measured length (*p* = 0.009). Sex and stocking history had no significant effect (*P* > 0.05) on TL and estimated age (Table 3).

**Table 2:** Estimates of Von Bertalanffy growth model parameters and standard error (SE) of asymptotic length (*L*_*inf*_), growth coefficient (*K*) and length-at-age 0 (*t0*) for the number of fish captured (*n*), average total length (mm) and mean age (year) for each population type. Unstocked lake populations are shaded.

Ecotype	Lake	*n*	Mean length (mm)	Mean age (year)	*L*_*inf*_	SE	*K*	SE	*t0*	SE
Piscivorous	Desert	22	438.3	11.5	760	44.66	0.08	0.001	-0.2	0.15
	Marguerite	32	491.5	11.8	673	27.27	0.11	0.008	-0.13	0.11
	Opeongo	26	485.3	10.5	583	18.1	0.15	0.01	0.31	0.12
	Cayamant	84	484.8	10.4	936	50.2	0.08	0.001	0.07	0.16
	Cèdres	77	539.9	10.6	773	16.03	0.12	0.001	0.07	0.07
Planktivorous	Antoine	28	417.4	11.4	532	17.52	0.13	0.009	-0.28	0.13
	Bondy	30	372.4	12.9	464	12.32	0.13	0.008	-0.43	0.12
	Shirley	18	385.8	12.8	479	16.54	0.13	0.01	-0.26	0.11
	Louisa	75	418.4	11.9	569	20.36	0.12	0.009	-0.59	0.14
	McFee	84	408.0	13.6	512	12.91	0.12	0.008	-0.59	0.14

SE: standard error

**Table 3:** Linear mixed effect models for the response variables of total length (mm) and estimated age (year). Columns present the estimates of differences (positive or negative) of group response variables with the intercept (piscivorous ecotype), standard error and *p*-values of the factor. *p*-values in bold are significantly different. The two-terms coefficients (separated by hyphens) represent the nested factors.

Coefficient	Response					
	Length (mm)			Age (year)		
	Estimate	SE	*p*-value	Estimate	SE	*p*-value
**Fixed parts**						
Intercept	473.06	16.63	**<0.001**	11.23	0.56	**<0.001**
Ecotype (planktivorous)	-87.07	22.63	**0.009**	0.94	0.76	0.26
Sex (male)	7.74	10.42	0.46	0.28	0.35	0.41
Stocking—piscivorous	8.18	24.28	0.75	-0.99	0.81	0.26
Stocking—planktivorous	24.90	22.61	0.31	0.56	0.75	0.48
**Random parts**						
*N*_*grp*_	10			10		
Observations	372			360		

SE: standard error

### Population genomics

**Fig 2:**
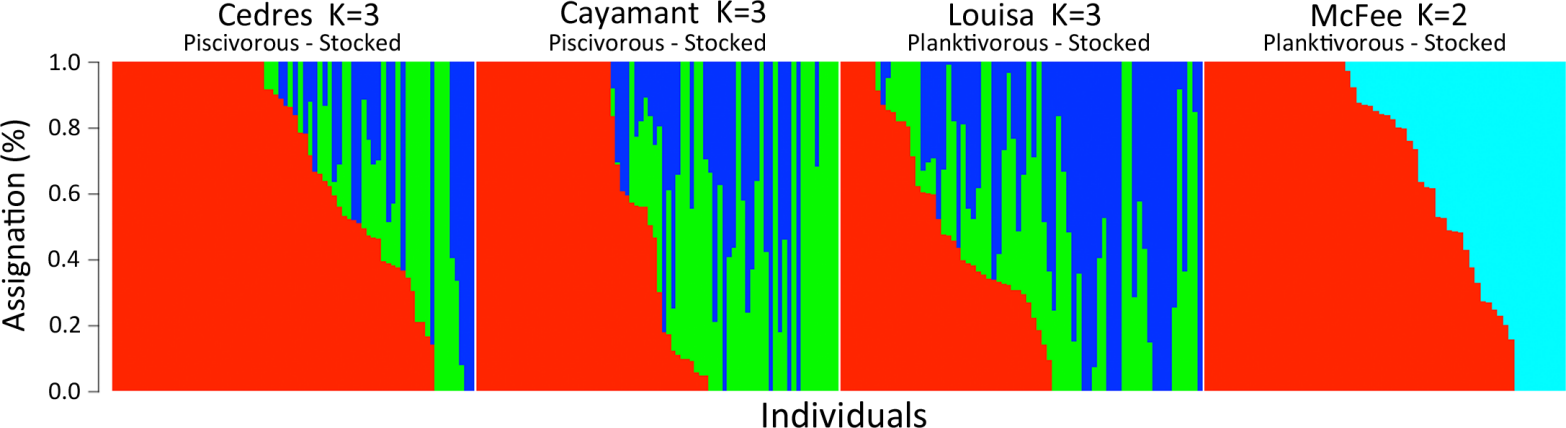
Admixture vertical plots for each stocked population. Vertical bars represent an individual and each different color corresponds to its assignment to one of the clusters (genetic ancestry): proportion of local ancestry (red), Blue Sea Lake (blue), Lake Trente et Un Milles (green) and Blue Sea in McFee Lake (aqua).

The total number of raw reads obtained by sequencing was 2 057 139 965, averaging 3 590 122 reads/individual. After filtering, we retained an average of 600 SNP markers (range = 553–700) for pairwise comparisons between the source and stocked populations. The low number of retained loci was expected given the trade-off of favoring the number of individuals retained in the analysis over loci. Yet, several hundred markers were sufficient to easily discriminate pure fish from fish of a different origin and their hybrids. Thus, in all cases, the most probable number of clusters was *K* = 2 when stockings were from only one source population and *K* = 3 when stockings were from two different source populations. This confirmed the accuracy of the stocking history records (Fig 2). As expected, all four stocked populations had fish from stocked, local and hybrid origins, with local individuals accounting for between 18% and 55% of the population sample (Table 4).

**Table 4:** Number of fish assigned to each genetic origin (percentage of within-population individuals), source of stocking (BS: Blue Sea Lake, 31M: Lake Trente et Un Milles) and the number of stocking events since 1900. Hybrids were individuals with ADMIXTURE local assignment (*Q* values) ranging from 10% to 90%.

Lake (*n*)	Local	Hybrid	Stocked	Sources	Events
McFee (67)	37 (55.2)	17 (25.4)	13 (19.4)	BS	2
Cèdres (75)	41 (54.7)	22 (29.3)	12 (16.0)	BS + 31M	9
Cayamant (79)	31 (39.2)	16 (20.3)	32 (40.5)	BS + 31M	17
Louisa (72)	13 (18.1)	33 (45.8)	26 (36.1)	BS + 31M	27

### Back-calculation and growth models

The regression of TL as a function of otolith radius for individual fish without considering ecotypes had a weaker fit (*L* = 0.38*S^0.972^, *R*^2^ = 0.59) than the ecotype-specific regressions; piscivorous (*L* = 0.27*S^1.03^, *R*^2^ = 0.72) and planktivorous (*L* = 0.811*S^0.85^, *R*^2^ = 0.62). There was a significant difference between the slopes of two regressions (ANCOVA, *F*_1,414_ = 68.25, *p* < 0.001). Therefore, back-calculations of length-at-age were made applying the ecotype-specific equations.

### Growth parameters estimations and comparisons

**Fig 3:**
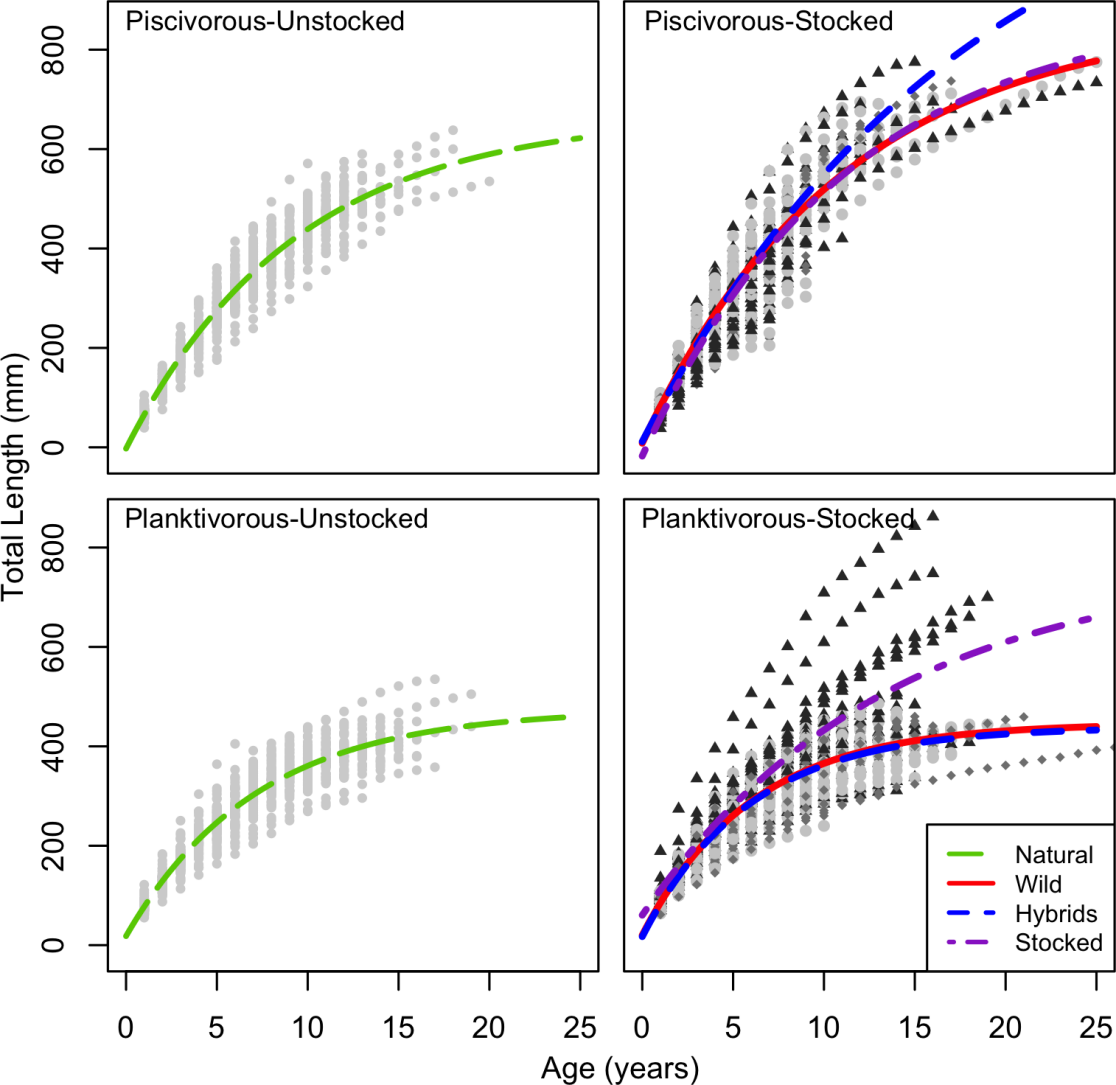
Individual growth curves by genetic origin within pooled unstocked and stocked populations of both ecotypes. Length-at-age of natural and local (light gray circles), hybrid (dark gray diamond) and stocked (black triangles) Lake Trout. Curves are global Von Bertalanffy growth models by genetic origin.

Estimates of the parameters for the population-specific Von Bertalanffy growth model (Table 2) showed the same trend as above: non-stocked piscivorous populations exhibited a larger *L*_*inf*_ than non-stocked planktivorous populations (Fig 3). Brody’s growth coefficients (*K*) were similar within ecotype, albeit more variable among piscivorous populations. Origins of regression (*t0*) were comparable among populations, varying between 0.07 and -0.6.

### Body condition index

Analyses of relative weight (*Wr*) in unstocked populations showed no significant difference between ecotypes (Welch *T*-test, *T*_120.3_ = 0.22, *p* = 0.82). There was also no significant linear relationship between *Wr* and TL (*t*-statistic_20,153_ = -1.45, *p* = 0.147) or age (*t*-statistic_20,146_ = -0.8, *p* = 0.425) in unstocked populations. Thus, variation in body condition was not influenced by body shape differences between ecotypes, length classes or age. As such, we considered that *Wr* could be used as a consistent proxy of the variation in body condition for Lake Trout of both ecotypes.

### Modeling the effect of stocking on growth parameters

Modeling the effects of ecotype, sex and genetic origin on individual growth parameters (*L*_*inf*_, ω) and the condition index (*Wr*) showed that sex had no significant effect on any of the response variables (Table 5). Ecotype had a significant negative effect on *L*_*inf*_, with planktivorous populations having a size about 300 mm smaller than piscivorous ones. Genetic origins had a significant negative effect in both ecotypes, showing a consistently larger *L*_*inf*_ for stocked individuals compared to local and hybrid fish. The greatest effect was linked to a hybrid origin in piscivorous populations; this negatively impacted *L*_*inf*_, driven mainly by the low *L*_*inf*_ of hybrids in Cayamant Lake. The only significant effect for the response variable ω (growth rate) was attributed to hybrid origins nested within the planktivorous population, which was significantly lower than stocked and local Lake Trout.

**Table 5:** Linear mixed effect models for response variables *L*_*inf*_, Omega and *Wr*. Columns present the differences (positive or negative) of group response variables with the model intercept (piscivorous ecotype), standard error (SE) and *p*-values of the factor. *p*-values in bold indicate significant differences (*p* < 0.05). The hyphen-separated coefficients represent nested factors.

Coefficient	Response								
	*L*_*inf*_			Omega			*Wr*		
	Estimate	SE	*p*-value	Estimate	SE	*p*-value	Estimate	SE	*p-value*
**Fixed parts**									
Intercept	882.98	48.47	**<0.001**	82.33	5.66	**<0.001**	91.91	5.89	**<0.001**
Ecotype (planktivorous)	-292.40	55.20	**0.03**	-6.98	7.30	0.44	2.31	8.07	0.82
Sex (male)	10.41	28.68	0.72	-0.03	2.32	0.99	-2.11	1.43	0.14
Hybrid–piscivorous	-216.47	73.73	**0.003**	5.44	5.96	0.36	1.03	3.67	0.78
Hybrid–planktivorous	-105.41	40.97	**0.02**	-9.75	3.30	**0.004**	5.91	2.03	**0.004**
Local–piscivorous	-122.78	59.84	**0.04**	2.48	4.85	0.61	1.65	2.99	0.58
Local–planktivorous	-120.26	40.83	**0.003**	-3.10	3.46	0.37	8.42	2.14	**<0.001**
**Random parts**									
*N*_*grp*_	4			4			4		
Observations	179			179			179		

**Fig 4:**
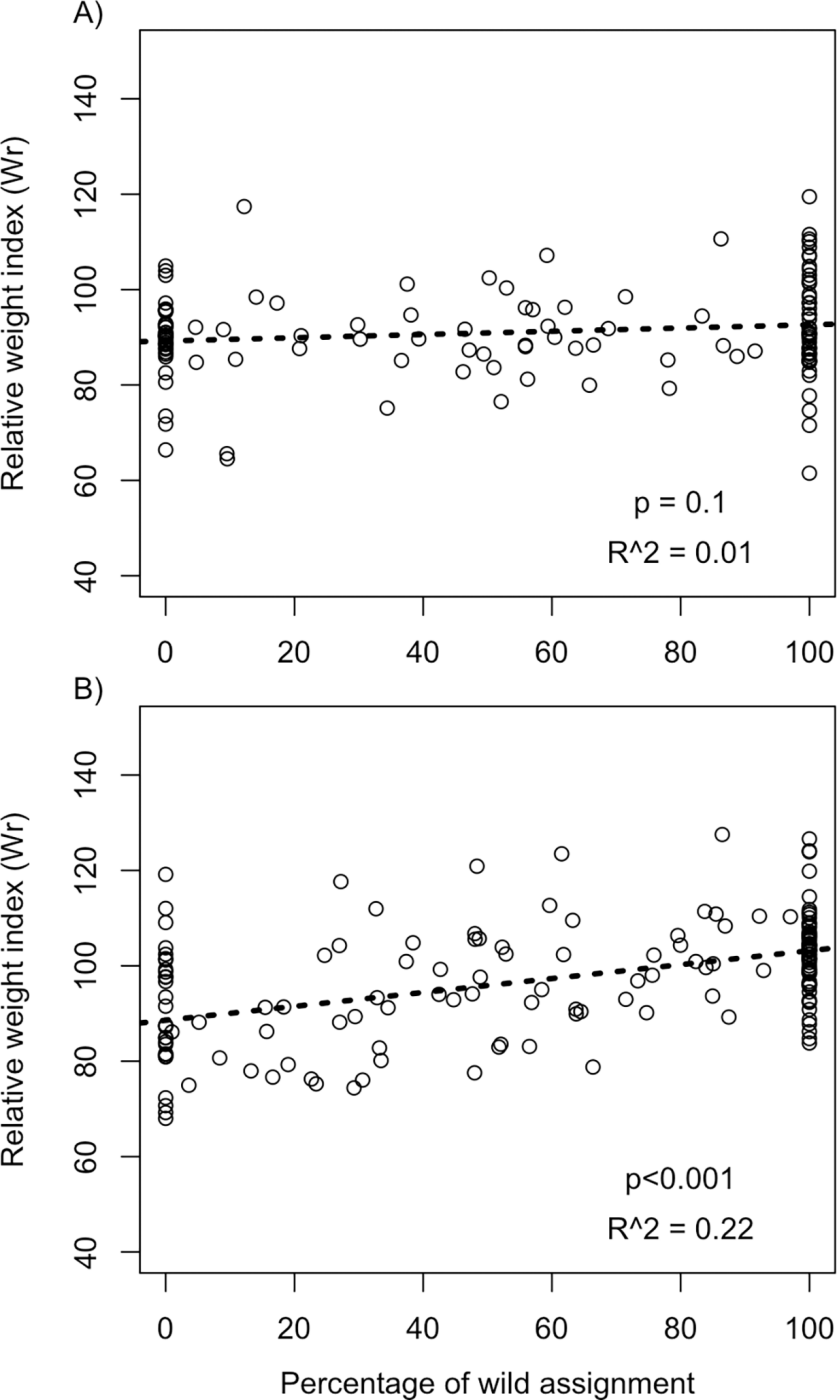
**Relative weight condition index (*Wr*) as a function of percentage of local assignment for (A) piscivorous and (B) planktivorous populations**.

Modeling of the body condition index showed that hybrid and local origins for planktivorous populations had a significant positive effect on *Wr* with local Lake Trout having the highest body condition index for this ecotype. Genetic origins had no significant effect on *Wr* in piscivorous populations, although there was a positive trend of an effect toward local fish having the highest condition index values. Finally, we observed a significant linear relationship between the percentage of local assignment (*Q*) of individual fish and *Wr* in stocked planktivorous (y = 0.15x + 88.6, *p* < 0.001, adj*R*^2^ = 0.22, *n* = 157) populations, but not in piscivorous (y = 0.03x + 89.1, *p* = 0.1, adj*R*^2^ = 0.01, *n* = 128) populations (Fig 4).

Individual growth curves were highly variable among stocked planktivorous populations (Fig 3). Approximately 80% of stocked fish exhibited planktivorous-like growth curves while the other 20% were characterized by a strikingly larger asymptotic length (TL > 500 mm). Growth rate and L_inf_ of those larger stocked Lake Trout were similar to those observed within piscivorous populations. Piscivorous-like stocked fish were significantly larger than either hybrids, local fish and the other 80% of stocked fish as of their first year of life (ANOVA on back-calculated length-at-age 1, *F*_3,133_ = 15.47, *p* < 0.001), and this difference persisted for the remainder of their life.

## Discussion

The objective of this study was to test whether ecotypes of supplemented and recipient Lake Trout populations could influence fish growth and condition. In both cases (supplementation using similar or contrasting ecotypes), asymptotic lengths of stocked individuals were greater than that of hybrids and local individuals. However, in planktivorous-stocked populations, most stocked individuals exhibited a planktivorous-like asymptotic length, whereas about 20% exhibited a piscivorous-like asymptotic length. Significant impacts on early life growth rates (omega) were observed only in hybrids within the stocked planktivorous population, the hybrids having lower growth rates than their congeners. The body condition index was only affected in populations stocked with contrasting ecotypes (planktivorous populations), marked by its positive relationship with the percentage of local assignment.

### Direct impact: Combined factors to foster a larger growth

The environmental conditions in hatcheries are much different than those of natural settings. Hatchery conditions include high fish density, low environmental variability, low predation risk and a consistent supply of food [13]. Even after one generation in hatchery, there should be strong directional selection for favorable traits in captivity, such as bold behavior and faster growth [18, 23, 76]. Accordingly, hatchery-reared salmonids generally exhibit higher growth rates and greater maximum lengths compared to their wild conspecifics [77, 78]. Higher growth rates should confer a competitive advantage to stocked fish and produce a displacement or decrease of local fish populations, contrasting use of spawning shoals [22] or higher gonadal productivity [13, 59]. On the other hand, hatchery-reared fish can exhibit significantly lower fitness than wild fish when selected traits are not adapted to new local conditions [9, 76].

In this study, higher asymptotic lengths of stocked individuals were observed for both ecotypes. This finding is evidence that even captive-bred broodstock can exhibit signs of directional selection from hatchery rearing at the expense of adapting to the natural environment [79]. However, whereas the length of stocked fish in piscivorous populations was homogeneous, the length in planktivorous populations was bimodal. The growth rate of Lake Trout is expected to stem from gene × environment interactions and be tightly linked to available prey [80, 81]. Whereas conditions likely to affect growth in stocked piscivorous lakes are concordant with the local growth regime (stocked piscivorous-originating individuals and large pelagic prey availability), this is not the case for stocked planktivorous lakes (stocked piscivorous-originating individuals and the absence of large pelagic prey). Individual fish are more likely to exhibit greater individual variability depending on their genetic background or individual traits and express variable responses for the latter.

### Genetic predisposition: Growing large at all cost?

Slow-growing planktivorous populations are typical of lakes where *Mysid* spp. or pelagic fish prey are absent [80, 82]. Even with their piscivorous genetic background, most stocked individuals in planktivorous lakes responded with growth and life histories typical of wild planktivorous individuals. In the absence of large energy-rich prey species (i.e., *Mysid* spp. or pelagic fish), the piscivorous life history, which requires caloric intake from those prey, can not be sustained [80]. Thus, a majority of fish exhibit a plastic response and adopt a phenotype that differs from that of their parents. However, about 20% of stocked fish can maintain a growth rate similar to rates observed in piscivorous populations. We hypothesize that this is due to cannibalism on juvenile conspecifics. Cannibalism is a life history trait that is occasionally observed within salmonid populations [83] but is generally considered as insignificant (< 2% of captured prey) in Lake Trout [45]. Based on the back-calculation data, since the time of stocking, putatively cannibalistic Lake Trout individuals were significantly larger (116.4 mm ± 27 mm) than their congeners (87.68 mm ± 11 mm). A previous meta-analysis of diet showed that > 50% of 150 mm (TL) lacustrine salmonids had fish prey in their stomach, and they readily fed on smaller-sized fish [84]. In our study, if the largest stocked individuals already fed on smaller juveniles at time of stocking, this would allow them to maintain a life-long size advantage. It is therefore likely that their piscivorous origin and possible hatchery selection for faster growth may have predisposed stocked fish to cannibalism, thereby increasing the occurrence of this phenomenon over the expected natural frequency.

### Impacts of hybridization on growth

Based on previous studies, we expected that the growth rate of hybrid Lake Trout would be intermediate between the parental lineages [13, 78, 85]. Here, we observed a significant difference only in the early life growth rate of hybrids from contrasting ecotypes (planktivorous/piscivorous hybrids). Those hybrids exhibited a significantly lower growth rate than their parental lineages. Given that the growth rate may be viewed as a proxy of individual performance and fitness [44, 86], the lower growth rate in piscivorous/planktivorous hybrids—compared to fish of other genetic origins—suggests a potential detrimental effect of hybridization. This effect could result from outbreeding depression [13, 85], although this remains speculative.

### Stocking large ecotypes: A suboptimal strategy?

The relationship between the relative weight condition index (*Wr*) and the proportion of local assignment (*Q*) highlights two important consequences of supplementing planktivorous populations with piscivorous fish. First, the stocked fish, even if exhibiting a planktivorous-like growth regime, are apparently maladapted for feeding on zooplankton or benthic prey. Indeed, given that the *Wr* index reflects the general health of fish [74], values below 100 are considered indicative of a potential shortage of trophic resources [87], depleted visceral fat [88], reduced growth rate [89] and low growth efficiency [90]. Similar observations have been made for other salmonids supplemented with domesticated populations [4, 24, 29]. This negative effect could be linked to hatchery selection; however, significant differences between the genetic origins in the mixed-effects model and the linear relationships were only observed in the supplemented planktivorous population. As all stocked fish originated from the same source population and hatchery, they should also have exhibited lower *Wr* in piscivorous populations. However this was not the case.

Additionally, part of this potential maladaptation may have a genetic basis given the linear relationship between body condition and the proportion of local assignment. Moreover, in supplemented planktivorous populations, the relationship was still significant when only hybrid individuals were considered (y = 0.21x + 85.9, *p* = 0.001, adj*R*^2^ = 0.13, *n* = 68), indicating that this relationship was not only driven by “pure” stocked and local individuals but also by hybrids of different generations. This suggests a polygenic architecture that underlies the phenotypic traits expressed as *Wr*. This observation is similar to the Burrishoole River experiment on hybridizing Atlantic Salmon (*Salmo salar*) where most phenotypic traits being intermediate between parental (wild and farm) values [85, 91].

### Study limitations

Given the available data, we could only compare lakes from one scenario of divergent hybridizing populations (piscivorous-planktivorous) and one scenario of a convergence of adaptive history (piscivorous-piscivorous). Other comparisons would have allowed for the testing of two additional hypotheses: i) stocking from planktivorous sources to planktivorous recipient populations minimizes the impacts on growth-related traits; and ii) stocking from a planktivorous source into a piscivorous recipient population induces higher growth rates for stocked individuals. These comparisons would have strengthened our conclusions regarding the impact of ecotypes on the outcomes of supplement stocking. However, the investigated scenarios remain very relevant as they are representative of most supplementation stocking programs in Quebec and Ontario, and these programs are also common elsewhere [6]. Finally, we emphasize that even if we observed minimal impacts from stocking with source and target populations of the same ecotype, more populations must be analyzed to confirm the effects of adaptive divergence between stocked and local populations on the growth-related outcomes of supplementation stocking.

## Conclusions

Supplementation stocking is rarely without impacts [6, 13, 27], and predicting the outcomes of supplementation remains a great challenge for management and conservation biology [92–94]. Our study showed that direct and indirect impacts varied depending on the similarity between the source and recipient stocks. Impacts of supplementation were generally small; however, the Lake Trout supplementation strategy in Quebec already incorporates multiple measures to minimize negative impacts (e.g., supplementation with F1 from a wild broodstock and supplementation at the fingerling or yearling stages). Nonetheless, we observed that supplementation stocking modified population growth and condition. We also noted detrimental effects associated with hybridization. Therefore, the similarities between source and supplemented populations must be considered within the decision framework of supplementation management. This can be assessed through simple metrics such as the population’s growth rate, maturation time or diet.

In the case of Lake Trout supplementation, ecotypes of source and recipient populations must be viewed as another risk factor that could lead to deleterious effects such as domestication [10, 11, 24, 29]. Ignoring a population’s genetic history and local adaptations can lead to a lower than expected return on investment by increasing cannibalism of juvenile Lake Trout and weakening the body condition of hybrid fish. On the other hand, our results show that supplementation stocking using more similar source/recipient population pairs has negligible ecological impacts. Finally, a precautionary approach in supplementation stocking practice should favor, whenever possible, use of local broodstock or at least a source population similar to the population being supplemented.

## Supporting information

S1 Appendix Standard Lake Trout sampling protocol. (DOCX)

S1 Table Lake Trout stocking history of studied lake. Table include year of the stocking event, line of fish used, age or stage at stocking (1+: 1 year old or F; fry), number of fish stocked, source population of stocking, density (number of fish/area of lake (ha)) and age of the survivals at sampling.
(DOCX)

S2 Table Parameters values for every module used for genotyping and loci filtering in the study. (DOCX)
