# Supplementary material for: Supplementation stocking of Lake Trout (Salvelinus namaycush) in small boreal lakes: Ecotypes influence on growth and condition
Source: PLoS One. 2018 Jul 12;13(7):e0200599. doi: 10.1371/journal.pone.0200599 (PMC6042763; doi:10.1371/journal.pone.0200599)
Supplement: S1 Appendix — (DOCX) [file pone.0200599.s001.docx]

S1 Appendix. Standard Lake Trout sampling protocol

Lake Trout were sampled at the end of summer (August to September), during the period of thermally stratified water column. Gill-net sampling effort was distributed across a semi-randomized grid of stations. The set of locations for each lake was randomly distributed where sampling depth strata were available. The sampling depth stratum is located at a maximum depth of 40 m in the overlap of a water temperature of 12 °C or less and a dissolved oxygen concentration of 5 mg*L^-1^ or more. Gill-net were 60 m long by 1.8 m high, and composed of eight 7.6 m panels of stretch mesh size ranging from 25 to 152 mm. Gill nets were deployed perpendicularly to the shore for an overnight effort ranging between 18 to 24 h and at least 75% of the gill-net length needed to be within the sampling depth strata.
