# Supplementary material for: Supplementation stocking of Lake Trout (Salvelinus namaycush) in small boreal lakes: Ecotypes influence on growth and condition
Source: PLoS One. 2018 Jul 12;13(7):e0200599. doi: 10.1371/journal.pone.0200599 (PMC6042763; doi:10.1371/journal.pone.0200599)
Supplement: S1 Table — Table include year of the stocking event, line of fish used, age or stage at stocking (1+: 1 year old or F; fry), number of fish stocked, source population of stocking, density (number of fish/area of lake (ha)) and age of the survivals at sampling. (DOCX) [file pone.0200599.s002.docx]

S1. Table Lake Trout stocking history of studied lake. Table include year of the stocking event, line of fish used, age or stage at stocking (1+: 1 year old or F; fry), number of fish stocked, source population of stocking, density (number of fish/area of lake (ha)) and age of the survivals at sampling.

| **Lake** | **Year** | **Line** | **Age or stage** | **Nb** | **Source** | **Density (nb/hec)** |
| --- | --- | --- | --- | --- | --- | --- |
| Cayamant | 1964 | F(1) | F | 15000 | Trente-et-un-Mille | 20.69 |
| Cayamant | 1965 | F(1) | F | 15000 | Trente-et-un-Mille | 20.69 |
| Cayamant | 1966 | F(1) | F | 15000 | Trente-et-un-Mille | 20.69 |
| Cayamant | 1987 | F(1) | 1+ | 4000 | Trente-et-un-Mille | 5.52 |
| Cayamant | 1988 | F(1) | 1+ | 4000 | Trente-et-un-Mille | 5.52 |
| Cayamant | 1989 | F(1) | 1+ | 1550 | Trente-et-un-Mille | 2.14 |
| Cayamant | 1991 | F(1) | 1+ | 1200 | Trente-et-un-Mille | 1.66 |
| Cayamant | 1993 | F(1) | 1+ | 2000 | Trente-et-un-Mille | 2.76 |
| Cayamant | 1995 | F(1) | 1+ | 3790 | Blue Sea | 5.23 |
| Cayamant | 1997 | F(1) | 1+ | 1500 | Blue Sea | 2.07 |
| Cayamant | 1999 | F(1) | 1+ | 4000 | Blue Sea | 5.52 |
| Cayamant | 2001 | F(1) | 1+ | 3600 | Blue Sea | 4.97 |
| Cayamant | 2003 | F(1) | 1+ | 5000 | Blue Sea | 6.90 |
| Cayamant | 2005 | F(1) | 1+ | 2400 | Blue Sea | 5.52 |
| Cayamant | 2007 | F(1) | 1+ | 2400 | Blue Sea | 4.97 |
| Cayamant | 2009 | F(1) | 1+ | 1500 | Blue Sea | 6.90 |
| Cayamant | 2011 | F(1) | 1+ | 2000 | Blue Sea | 3.31 |
| Mc Fee | 1990 | F(1) | F | 1000 | NA | 10.75 |
| Mc Fee | 2001 | F(1) | 1+ | 2000 | Blue Sea | 21.51 |
| Louisa | 1956 | F(1) | F | 2000 | NA | 4.55 |
| Louisa | 1958 | F(1) | F | 10000 | NA | 22.73 |
| Louisa | 1969 | F(1) | F | 5000 | Mégantic | 11.36 |
| Louisa | 1970 | F(1) | F | 3000 | Massawippi | 6.82 |
| Louisa | 1975 | F(1) | 1+ | 1500 | Waconichi | 3.41 |
| Louisa | 1976 | F(1) | 1+ | 1500 | Trente-et-un-Mille | 3.41 |
| Louisa | 1977 | F(1) | 1+ | 1500 | Trente-et-un-Mille | 3.41 |
| Louisa | 1978 | F(1) | 1+ | 1250 | Trente-et-un-Mille | 2.84 |
| Louisa | 1981 | F(1) | F | 3000 | Trente-et-un-Mille | 6.82 |
| Louisa | 1986 | F(1) | 1+ | 2000 | Trente-et-un-Mille | 4.55 |
| Louisa | 1987 | F(1) | 1+ | 4000 | Trente-et-un-Mille | 9.09 |
| Louisa | 1988 | F(1) | 1+ | 3000 | Trente-et-un-Mille | 6.82 |
| Louisa | 1988 | F(1) | F | 2000 | Trente-et-un-Mille | 4.55 |
| Louisa | 1989 | F(1) | F | 7000 | Trente-et-un-Mille | 15.91 |
| Louisa | 1996 | F(1) | 1+ | 1250 | Trente-et-un-Mille | 2.84 |
| Louisa | 1998 | F(1) | 1+ | 950 | Trente-et-un-Mille | 2.16 |
| Louisa | 1998 | F(1) | 1+ | 1000 | Trente-et-un-Mille | 2.27 |
| Louisa | 1999 | F(1) | F | 500 | Trente-et-un-Mille | 1.14 |
| Louisa | 1999 | F(1) | F | 1200 | Trente-et-un-Mille | 2.73 |
| Louisa | 2000 | F(1) | 1+ | 3000 | Trente-et-un-Mille | 6.82 |
| Louisa | 2000 | F(1) | 1+ | 1250 | Trente-et-un-Mille | 2.84 |
| Louisa | 2001 | F(1) | 1+ | 2000 | Trente-et-un-Mille | 4.55 |
| Louisa | 2002 | F(1) | 1+ | 2000 | Trente-et-un-Mille | 4.55 |
| Louisa | 2003 | F(1) | F | 3000 | Trente-et-un-Mille | 6.82 |
| Louisa | 2004 | F(1) | 1+ | 3000 | Trente-et-un-Mille | 6.82 |
| Louisa | 2006 | F(1) | 1+ | 1400 | Trente-et-un-Mille | 3.18 |
| Louisa | 2006 | F(1) | F | 3000 | Trente-et-un-Mille | 6.82 |
| Cèdres | 1964 | F(1) | F | 4488 | NA | 15.91 |
| Cèdres | 1974 | F(1) | F | 2000 | NA | 7.09 |
| Cèdres | 1995 | F(1) | 1+ | 3400 | Blue Sea | 12.06 |
| Cèdres | 1997 | F(1) | 1+ | 2600 | Blue Sea | 9.22 |
| Cèdres | 1999 | F(1) | 1+ | 3000 | Blue Sea | 10.64 |
| Cèdres | 2001 | F(1) | 1+ | 4000 | Blue Sea | 14.18 |
| Cèdres | 2003 | F(1) | 1+ | 5000 | Blue Sea | 17.73 |
| Cèdres | 2005 | F(1) | 1+ | 2600 | Blue Sea | 9.22 |
| Cèdres | 2011 | F(1) | 1+ | 3500 | Blue Sea | 12.41 |
