# Supplementary material for: Supplementation stocking of Lake Trout (Salvelinus namaycush) in small boreal lakes: Ecotypes influence on growth and condition
Source: PLoS One. 2018 Jul 12;13(7):e0200599. doi: 10.1371/journal.pone.0200599 (PMC6042763; doi:10.1371/journal.pone.0200599)
Supplement: S2 Table — (DOCX) [file pone.0200599.s003.docx]

S2 Table. Parameters values for every module used for genotyping and loci filtering in the study

| **Modules** | **Parameters** | **Values** |
| --- | --- | --- |
| *ustacks* | Minimum depth coverage (m) | 3 |
|  | Maximum distance between stacks (M) | 2 |
|  | Maximum distance to secondary read (N) | 4 |
| *cstacks* | Number of mismatch allowed (n) | 1 |
| *sstacks* | *Default parameters* |  |
| *stacks_rx* | Minimum log-likelihood to keep a locus | 10 |
| *cstacks_rx* | Number of mismatch allowed (n) | 1 |
| *Populations* | Minimum percentage of individuals required in a population to process a locus (r) | 0.5 |
|  | Minimum number of populations a locus must be present | 1 or 2 |
|  | Minimum stacks depth at a locus (m) | 5 |
|  | Minimum minor allele frequency (*maf*) before calculating Fst at a locus (a) | 0.001 |
| *Locus*  *Filtering* | Minimum depth to keep a genotype | 5 |
|  | Maximum allelic imbalance | 8 |
|  | Minimum percentage of presence of a locus in a population | 70 |
|  | Minimum *maf* that must be respected in all population to retain locus | 0.01 |
|  | Minimum *maf* that must be found in at least one population to retain locus | 0.05 |
|  | Maximum proportion of heterozygous individuals | 0.7 |
|  | Minimum Fis value | 0.7 |
|  | Maximum Fis value | 0.7 |
|  | Maximum number of SNP per locus | 10 |
